# Physiotherapy assessment for female urinary incontinence

**DOI:** 10.1007/s00192-020-04251-2

**Published:** 2020-03-03

**Authors:** B. Berghmans, M. R. Seleme, A. T. M. Bernards

**Affiliations:** 1 Pelvic care Center Maastricht, Maastricht University Medical Center, P.O. Box 5800, 6202 az Maastricht, The Netherlands; 2 abafi-HOLLAND, Maastricht, The Netherlands; 3 Faculdade Inspirar, Curitiba, Brazil; 4 Department of Physiotherapy, Centre for Musculoskeletal Rehabilitation, HAN University of Applied Sciences, Nijmegen, The Netherlands

**Keywords:** Female, Urinary incontinence, Assessment, (Pelvic) Physiotherapy, Diagnostic, Initial management, Psychometric measurement

## Abstract

**Introduction and hypothesis:**

As noted in the 6th International Consultation on Incontinence (ICI) chapter “Initial Management of Urinary Incontinence in Women” recommendations call for including physiotherapy as a first-line therapy.

**Methods:**

Building on this, checking available scientific evidence and using the International Classification of Functioning, Disabilities and Health, the following represents a holistic physiotherapist approach for initial evaluation of the health problem of urinary incontinence.

**Results:**

This paper proposes a teaching module for every relevant health care professional dealing with the assessment of adult female urinary incontinence, focusing on optimal patient selection and appropriate treatment choice.

**Conclusion:**

The assessment stage involves the explicit decision as to whether “physiotherapy” is the treatment indicated for the patient, based on the findings of the physiotherapy assessment and supplemented by any medical information that accompanied the referral and evaluation.

## Introduction

In this paper the main focus is on physiotherapy. However, it is intended as a proposal and starting point to discuss and develop a teaching module for every relevant health care professional dealing with the assessment and treatment of female urinary incontinence (UI).

As noted in the 6th International Consultation on Incontinence (ICI), recommendations for the *initial* management of adult female UI are based on a medical diagnosis that does not always take into account co-morbidities or functional limitations that can affect outcomes [1]. This means that sometimes a patient with unclear and/or underlying pathology that is not amendable to physical therapy is referred to physiotherapy for treatment. Without taking these issues into consideration, therapy is often disappointing and has inadequate results.

A holistic structured and systematic physiotherapy assessment in women with UI may help to improve patient selection and identify those who will benefit most from physiotherapy.

## Materials and methods

Two out of the three authors of this paper (BB, ATMB) were originators of the evidence-based Royal Dutch Society for Physiotherapy (KNGF) guidelines for UI, which have been developed, implemented and published in multiple languages [2]. These guidelines have been extensively checked with relevant literature and available national and international guidelines [2]. For this paper we again performed a systematic literature search (see Appendixes 1, 2) between 1998 and November 2019 in the following databases: PUBMED, MEDLINE, EMBASE, CINAHL, PEDro, and the Cochrane database. Publications about the methods of diagnosing stress, urgency, and mixed UI and about their reliability, validity, and feasibility of assessment for physiotherapy in routine practice, were retrieved. We used the search term “urinary incontinence” with the following keywords: “diagnosis,” “assessment,” “guidelines,” “ informed consent,” “predictors,” “intra-abdominal pressure,” “low back pain,” “COPD,” “pelvic floor assessment,” “strength,” “endurance,” “pad test,” “diary,” “hygiene,” “manual muscle testing,” “vaginal squeeze pressure,” “palpation,” “digital assessment,” “manometry,” “pelvic floor function,” “pelvic floor assessment,” “EMG,” “validity,” “reliability,” “sensitivity,” “specificity,” “quality-of-life,” “posture,” “respiration,” and “psychometric measurement.”

After screening, nine relevant guidelines and six systematic reviews were identified and analyzed (see Appendix 3), cross-checking the findings with the levels and grades of evidence from recommendations of the 6th International Consultation on Incontinence [1], utilizing the International Consultation on Urologic Diseases—Evidence Based Medicine grades. According to this, the grade of recommendation in the area of “initial assessment,” which is mostly the case in conservative management, has little evidence outside of “expert opinion,” which is a recognized limitation (Level 4, Grade D) [3].

The assessment process, which is used to optimize patient selection and to formulate a specific treatment plan, investigates the nature of the underlying disorders and co-existing functional limitations that affect UI and its severity (using terminology of the International Classification of Functioning, Disability and Health (ICF; Table 1) [4]. These are examined in the context of whether the underlying disorders and/or any identified unfavorable prognostic factors are modifiable by physiotherapy.

**Table 1:** Definitions of the International Classification of Functioning Terms

Category	Description
Impairment	Loss or abnormality of psychological, physiological, or anatomical structure or function at organ level. With respect to the classification of disorders in the storage and voiding of urine and feces, this means the impairment stress incontinence or detrusor overactivity
Disability	Restriction or loss of ability of a person to perform functions/activities in a normal manner. With respect to the classification of disabilities of voiding and stool, this means the disability involuntary loss of urine
Restriction in participation	Disadvantage due to impairment or disability that limits or prevents fulfilment of a normal role (depends on age, sex, socio-cultural factors) for the person

Source: WHO Publication [4]

## Basic recommendations

### “Red and yellow flags”

In cases in which there has been no prior *medical* assessment, before starting the physiotherapy assessing process, the physiotherapist must screen for pathological conditions that would require medical consultation. The physiotherapist should pay particular attention to the following symptom patterns, which may be indicated as “red flags” (level 4, grade D) [2, 3, 5]:

- Unexplained incontinence, for instance, insensible or continuous incontinence, as this may represent a vesico-vaginal fistula
- Sudden onset incontinence in an elderly patient
- Pain while urinating
- Hematuria
- Urinary retention/overflow incontinence
- Signs of vaginal/anal inflammation
- (Persistent or recurrent) infections/urinary tract infection
- Prolapse below the introitus
- Fever
- Signs of general malaise
- Severe loss of weight
- Fistula
- Suspected undiagnosed underlying neurological disorder
- Rectal bleeding

The physiotherapist should focus on recognizing symptom patterns and identifying possible “yellow flags,” which would require thorough consideration of the physiotherapist and may first require medical consultation before moving on to the assessment [2]. These would include psychological/sexual trauma, which makes a vaginal examination difficult. Identification of yellow flags in the context of local and/or general inhibiting factors for improvement or recovery is also warranted in the case of:

- Uncontrolled cardiovascular diseases
- Back, pelvic, and/or hip dysfunction
- Diabetes
- COPD
- Sexual problems
- Use of medication (diuretics, sympathomimetics/sympatholytics, parasympathomimetics/parasympatholytics

Once the pattern indicates UI, without any other abnormal findings of red or yellow flags, the physiotherapy assessment can be initiated.

### Hygiene and ethical requirements

Hygienic procedures, the physiotherapist’s behavior, and actions during the complete physiotherapy assessment process should conform to standards and protocols, in line with local policy, rules, and requirements [2, 6].

### Informed consent

The patient should be informed, both verbally and—as required in some countries—in writing, about the aims and methods of the physiotherapy assessment process; for example, a vaginal and/or anorectal assessment might be required [7]. If an internal examination is required, documented verbal or signed consent (according to local policy) should be obtained.

### Instruction of the patient beforehand

Where possible, the patient is instructed to come to the clinic with a comfortably full bladder (because of the need for provocation tests, e.g., cough stress test, during the physical examination) [7, 8].

### Components of the physiotherapy assessment

Information is obtained by means of a thorough history-taking, the patient’s self-report, validated questionnaires, frequency–volume chart or bladder diaries, and the therapist’s own physical examination of the patient.

Relevant factors such as type, frequency, severity, precipitating factors, social impact, effect on hygiene and quality of life, the measures used to contain the leakage, and whether or not the individual seeks or desires help should be specified (level 4, grade D) [3].

Using the International Classification of Functioning, Disability and Health, dealing with the *consequences* of the health problem UI [4], the physiotherapist will investigate the following levels:

1. Organic level, i.e., impairments in physiological function or anatomical structures, e.g., weakness of the pelvic floor, tears of connective tissue, and/or pelvic floor muscle (PFM); straining with defecation.
2. Local level, e.g., using protective products.
3. Personal level, i.e., disabilities, limitations, such as not being able to lift a baby or stand up from a seat without involuntary urine loss.
4. Socio-cultural level, i.e., restriction in participation, such as work, sport activities or social engagements, no longer attending activities without the fear of urine loss, bad odor, etc.
5. Contextual factors, external and personal factors, e.g., unable to attend visits, poor motivation, etc.

All these consequences are the basis for and the key elements of a plan of intervention [2].

## History-taking, self-report, and questionnaires

A goal-oriented and systematic history-taking involves good medical/paramedical communication, and rapport with the patient. The therapist asks standardized, in-depth questions about the type, amount, timeline, and precipitating and aggravating factors of urinary leakage to determine the type of incontinence that the woman is experiencing (level 4, grade D) [3].

There are short questionnaires that may be used to assist with this clinical diagnosis (level 3, grade C). The ICIQ-UI SF is a fully, validated, reproducible, grade A, four-item questionnaire that can be used to obtain a brief, yet comprehensive, summary of the level, impact, and perceived cause of symptoms of incontinence [9]. The 3IQ test (Table 2) allows rapid distinction of the main types of UI: stress urinary incontinence (SUI), urgency urinary incontinence (UUI), and mixed (both stress and urgency) urinary incontinence (MUI) [10, 11]. The 3IQ test (Table 2) is a useful, validated measurement instrument that can establish the presence of SUI with a sensitivity of 0.86 (95% CI = 0.79–0.90) and a specificity of 0.60 (95% CI = 0.51–0.68), UUI with a sensitivity of 0.75 (95% CI = 0.68–0.81) and a specificity of 0.77 (95% CI = 0.69–0.84), and MUI with a sensitivity and specificity of 0.58 (95% CI = 41–74) and 0.64 (95% CI = 56–72) respectively [10].

**Table 2:** The 3IQ test [10]

Questionnaire questions	Definitions of type of urinary incontinence based on responses to question 3
1. During the last 3 months, have you leaked urine (even a small amount)? Yes (please continue with questions 2 and 3) or no (questionnaire completed)	
2. During the last 3 months, did you experience any involuntary loss of urine: (check all questions that apply)	
a) When you were performing some physical activity, such as coughing, sneezing, lifting, or exercise?	
b) When you had the urge to empty your bladder, but you could not get to the toilet fast enough?	
c) Without physical activity and without a sense of urgency?	
3. During the last 3 months, did you experience involuntary loss of urine most often: (check only one)	
a) When you were performing some physical activity, such as coughing, sneezing, lifting, or exercise?	a) Most often with physical activity ➞ stress-only or stress-predominant urinary incontinence
b) When you had the urge to empty your bladder, but you could not get to the toilet fast enough?	b) Most often with the urge to empty the bladder ➞ urge-only or urge-predominant (urinary) incontinence
c) Without physical activity and without a sense of urgency?	c) Without physical activity or sense of urgency ➞ other cause
d) About equally as often with physical activity as with a sense of urgency?	d) About equally with physical activity and sense of urgency ➞ mixed (urinary) incontinence

## Identifying the severity of the health problem

It is important to assess the severity of the patient’s health problem by identifying the existence and extent of impairments (local level), limitations of activities/disabilities (personal level) and restrictions to participation (socio-cultural level) [4]. The severity of the health problem is determined by the frequency and magnitude of the involuntary urine loss, the use of incontinence absorptive products and the consequences for everyday life, including work, sports, housekeeping activities, family and social life, and sexuality.

### PRAFAB questionnaire

An instrument that assesses both urine loss and its impact on the patient is the validated PRAFAB questionnaire [12]. This questionnaire has a high internal consistency and construct validity and got the highest ranking from the ICI (level 1, grade A) [3]. The PRAFAB questionnaire (Table 3) measures the severity of urine loss in terms of the use of absorptive products, (*Pr*otection) the magnitude of the urine loss (*A*mount), and the number of times urine is lost (*F*requency). In addition, it records the impact of the urine loss more subjectively, such as the way the patient adjusts to the urine loss in everyday life (*A*djustment) and the consequences of the incontinence for the patient’s self-image (*B*ody image). The PRAFAB questionnaire thus combines key objective and subjective aspects of the incontinence problem. The questionnaire measures two separate domains by means of a “leakage severity scale” and a “perceived impact scale” [13].

**Table 3:** The PRAFAB questionnaire score*

Category	Options
Protection	1. I never use protection for urine loss
	2. I sometimes use protection, or I have to change my underwear because of urine loss
	3. I normally use protection, or change my underwear several times a day because of urine loss
	4. I always have to use protection because of urinary incontinence
Amount	1. The amount of urine loss is just a drop or less
	2. Sometimes I lose a trickle
	3. The loss of urine is so much that it noticeably wets my protection or clothes
	4. The loss of urine is so much that my protection is soaked or leaks
Frequency: involuntary loss of urine occurs	1. Once a week or less
	2. More than once but less than three times a week
	3. More than three times a week, but not every day
	4. Every day
Adjustment: implications of urine loss	1. I am not hampered in my daily activities
	2. I have stopped some activities, such as some sports and physically demanding activities
	3. I have stopped most physical activities that caused involuntary loss of urine
	4. I almost never go out
Body (or self) image	1. I am not bothered by my urine loss
	2. I think urine loss is annoying and troublesome, but I am not greatly bothered by it
	3. Urine loss makes me feel dirty
	4. I am disgusted by myself because of my urinary incontinence
Total score	

The PRAFAB questionnaire is validated in Dutch. Psychometric testing of the English version is not (yet) performed. Nevertheless this questionnaire is provided in English to give the readers an insight into the items and scoring system (minimum-maximum = 5–20 points; range 16 points)

### Urgency questionnaire

**Fig. 1:**
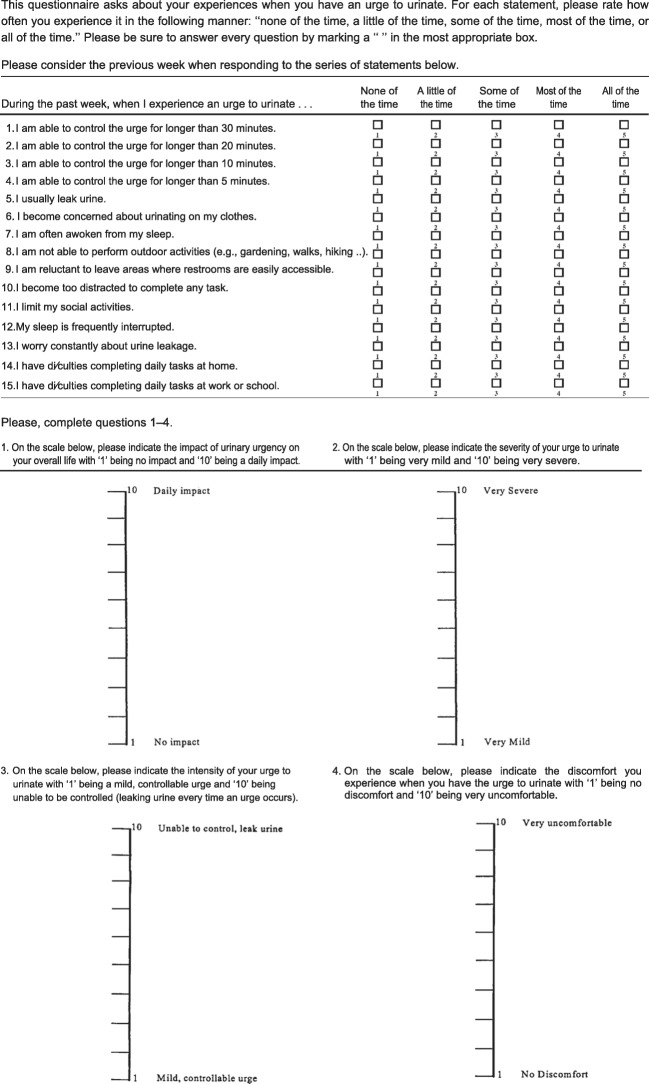
Urgency questionnaire

Specifically for UUI, several questionnaires can be used [3]. The Urgency Questionnaire (Fig. 1) is reported to be a reliable, valid, and responsive instrument, has been allocated grade A and recommended by the ICI (level 1, grade A). It utilizes four visual analog scale (VAS) items to assess the overall health-related quality of life, impact, severity, intensity, and discomfort of UUI respectively, and 15 Likert scale items [14].

Particular attention should be paid to the patient’s own ideas and views (“illness beliefs”) about their incontinence, its possible causes and consequences, the chances of recovery, what they can do about it themselves, what can be done by the therapist, and what contribution or role can be expected of others. Illness beliefs are individual and acquired during life and during the course of an illness and can have a favorable or adverse effect on the prognosis in terms of recovery [3].

Beliefs may partly determine the type of intervention that can be used and can affect expectations of both the patient and the physiotherapist (level 3, grade C) [15].

## Identifying etiological and prognostic factors for success

In addition to acquire information regarding if and to what extent (the consequence of) unfavorable factors can be modified by physiotherapy, history-taking should also contain questions about:

1. The nature and etiology of the underlying cause of UI, e.g., the woman’s obstetric history
2. Any factors that may influence the course of UI and the recovery process:
  Local factors impairing improvement/cure, e.g., co-existing apical prolapse [16], and/or increasing strain on the pelvic floor, e.g., caused by excessive straining during defecation
  General factors impairing the recovery process after pelvic floor injury, including factors relating to the patient’s physical and/or psychological condition, e.g., psychological distress, obesity or poor physical health [3, 17]

## Additional testing for type and severity of urinary incontinence

### Bladder diary

A frequency-volume chart records the time of each micturition and the volume voided, a bladder diary additionally includes fluid intake, pad usage, number of incontinence episodes, and the degree of incontinence [18]. Information about a number of variables relating to micturition by day and night, involuntary urine loss, and activities during which the incontinence occurs, as well as fluid intake and urgency episodes can be obtained.

The following variables are systematically recorded, preferably covering at least 3 consecutive days that are representative of the patient’s daily activity patterns, for example, 2 working days and 1 weekend day (level 3 grade C) [3].

- When, what, and how much the patient drinks
- The level of urgency
- The amount and timing of micturition
- The times when involuntary urine loss occurs and the amounts of urine lost, subjectively

The bladder diary may support the assessment of both type and severity of UI. A patient suffering from SUI usually has a normal voiding frequency (less than or equal to 8 times in 24 h) [19] and bladder volume, has mean micturition between 200 and 400 cc/void [20], but with neither urgency nor nocturia. The patient with stress incontinence might report losing small amounts of urine during exertion, whereas a patient with UUI experiences urgency, has higher (more than eight times in 24 h) voiding frequency, may experience nocturia, typically loses larger volumes (even up to complete emptying of the bladder). If the patient voids less than 150 ml of urine during micturition, this might suggest a reduced functional capacity of the bladder.

### Physical examination

The physical examination consists of inspection at rest and inspection during movement, digital palpation, and functional examination, and has the following objectives:

- Assessing whether and to what extent other parts of the musculoskeletal system are hampering the function of the PFMs
- Assessing the extent of voluntary and involuntary control over the pelvic floor
- Assessing PFM function
- Identifying any local (e.g., apical prolapse) and generally unfavorable prognostic factors (e.g., postural dysfunction)

During the physical examination, the patient’s dignity and comfort must be maintained at all times. During the assessment, the physiotherapist watches for signs of non-verbal communication, maintaining—whenever possible—eye contact and watching for guarding and breath holding and any signs of pain.

#### General physical examination

General physical examination can be used to identify signs of reduced pelvic floor toughness. The severity of the UI is dependent on the condition of the pelvic floor and is influenced by the patient’s respiration, movement patterns, and general physical and psychological status (level 4, grade C) [7, 8]. Therefore, it is important to not only examine the patient locally (i.e., their abdominal and pelvic regions) but also to assess the patient’s overall condition. For instance, obesity is an unfavorable prognostic factor for recovery and can be assessed using BMI measurement [21].

The physiotherapist should assess whether and to what extent other parts of the musculoskeletal system are hampering the function of the PFM. The physiotherapist should inspect and observe [2, 22]:

1. Patient’s sitting and standing posture (this may have relevance to a patient’s urethral angle, anorectal angle, abdominal pressure, and toileting behavior), including the spinal curvature, pelvic torsion or position, rib position, shoulder symmetry, tension of muscles such as the abdominal, neck, and calf muscles.
2. Respiration (breath holding and vocal behavior): rib movement, activity of the respiratory muscles, abdominal activity, tensed or relaxed?
3. Joint mobility of the hips, pelvis, coccyx, spinal column, movement patterns, tonicity of the surrounding musculoskeletal tissues.

#### Interpretation of the general physical examination

A strong relationship is described between lower back pain on the one hand and UI and respiratory dysfunction on the other, as the consequence of a limited ability to sufficiently integrate trunk muscle function in the regulation of posture and respiration, as well as continence [22–25].

The need to carry out a general examination is based on studies investigating pelvic floor impairments originating from other parts of the musculoskeletal system. The role and impact of these interactions have to be viewed with caution, as the methodological quality of these studies is at the most moderate (level 3, grade C) [2]. Studying and analyzing its role and impact requires further and more detailed research [2].

### Pelvic examination and vaginal assessment

The patient is in a semi-supine position, with knees bent and spread, and the upper body tilted at 35°. If possible, the end of the treatment table should be tilted at 30° so that the patient can rest her feet with anteflexion of the ankles. The physiotherapist should wear non-latex, non-sterile gloves and apron and apply rigorous infection control.

Using procedures in accordance with local protocols, the physiotherapist will inspect:

- The upper thighs, the skin of the perineal region and the outer labia: any skin irritations (which indicate more or less permanent moistness or use of unsuitable UI products) are noted.
- The perineum and the entrance and distal part of the vagina: this requires spreading the outer and inner labia; gel or lukewarm water may be used; any rupture scars or scars caused by episiotomy, or atrophy of the PFMs are noted, the urethral opening located, the entrance to the vagina inspected; any signs of vaginitis (red and dry instead of pink and moist), any discharge that is abnormal of offensive in smell (leukorrhea) is noted and fungal infection should be excluded).
- The vagina: any signs of anterior or posterior vaginal wall defects, uterine prolapse, tissue quality (vaginal atrophy) are noted; neurological examination (clitoris reflex and dermatomes), stress cough test [8].
- The anus: any signs of hemorrhoids, anal gaping at rest or fissures are noted; neurological examination (anal wink reflex and dermatomes).

The patient may need to be in the left lateral position if the anus cannot be observed in the supine position.

Next, the physiotherapist should ascertain PFM function and to what extent the patient has voluntary control over and awareness of her pelvic floor. Exercising or training the PFMs can only be successful if the patient is able to voluntarily contract and relax her PFMs.

Voluntary contraction of the PFMs means that the patient is able to contract them on demand. PFM relaxation should be tested after a contraction. Therefore, the investigator should always start with a contraction and then ask for relaxation. This is perceived as the cessation of contraction.

Pelvic examination by inspection provides information about whether an inward movement of the PFMs is visible on contraction, whether any co-contraction and relaxation is visible, and whether movement of the perineum is visible on coughing and straining [2, 26].

Before assessing the functionality of the pelvic floor by digital palpation, the presence of pain intra-vaginally (not uncommon in pelvic pain or neuropathy) is assessed by palpating the walls of the vagina with the index finger, starting at the 6 o’clock position (which is closest to the coccyx at the level of the hymnal remnants of the vagina and slowly moving the finger toward the 9, 12, 3, and again the 6 o’clock position at the same level, followed by another round a little deeper inside the vagina and so on just like a corkscrew, going deeper and deeper. Using a numerical rating scale, any pain is rated. Conclusions are made whether or not a digital palpation is possible and can be tolerated by the patient.

If palpation is possible, vaginal or rectal palpation, using one (index) or two (index and middle) fingers, enables the therapist first to assess the PFM resting tone. Muscle tone may be altered in the presence or absence of pain. However, this assessment is hampered by the fact that there is no single accepted or standardized way of measuring muscle tone, and there are no normative values for the term normal tonus, hypertonus, and hypotonus [27]. The physiotherapist may determine, in relation to a resting tone, hypertonus as abnormally elevated contractile activity and hypotonus as abnormal reduced contractile activity [27]. In the future, it is hoped that besides digital palpation of resting tone, objective measures and cut-off points of PFM elasticity can be developed, both for research and clinical use, to increase the validity and repeatability of the assessment of tonus [28].

Vaginal or rectal palpation also enables the therapist to evaluate the correct performance of a voluntary and an involuntary (during coughing or straining/Valsalva) PFM contraction and relaxation [29]. A valid contraction must be perceived as an encircling, elevating (inward) movement and tightening sensation around one or two palpating fingers. According to the ICS terminology, the contraction may be categorized as being “absent,” “weak,” “normal” or “strong” [30].

The strength of the PFM contraction is graded as:

- Absent, no palpable response
- Weak, i.e., weak contraction (short contraction, no palpable closing movement)
- Normal, i.e., moderate contraction (closing and cranio-ventral movement against light resistance palpable)
- Strong, i.e., good contraction (a powerful closing and cranio-ventral movement against firm resistance palpable)

After a PFM contraction, (in-)voluntary “relaxation” means that the PFM tone should at least return to its resting state. The ICS recommends rating (in-)voluntary relaxation as “absent,” “partial,” “complete,” or “delayed” [27, 30].

**Fig. 2:**
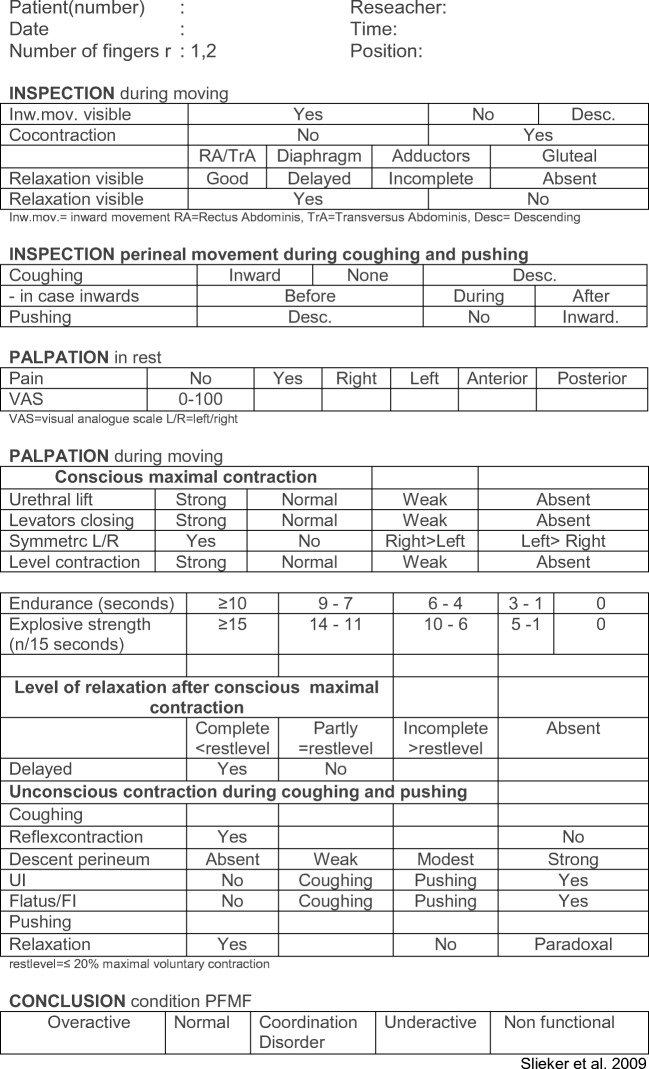
Pelvic floor muscle functional assessment. *UI* urinary incontinence, *FI* fecal incontinence

To evaluate PFM function, the following assessment schedule and interpretations of assessment have been described in the “Royal Dutch Society for Physiotherapy (KNGF) practice guidelines for patients with stress urinary incontinence” (Fig. 2) [2, 26]:

- Assess whether the patient is able to voluntarily contract and relax the pelvic floor, and evaluate the performance.
- Assess the presence and correctness of the voluntary contraction and relaxation of the PFMs.
- Assess the presence and correctness of involuntary contraction of the PFMs associated with a sudden increase in intra-abdominal pressure (forceful coughing) and subsequently whether a voluntary PFM correctness without and after instruction can be maintained during coughing.
- Assess the presence and correctness of involuntary relaxation of the PFMs during straining.
- Observe the voluntary contraction and relaxation of the PFMs in relation to the ability to isolate a PFM contraction with only appropriate rather than excessive co-activation of the abdominal muscles [31].
- Quantify the strength, endurance, and explosive strength of the PFMs using manual muscle tests, such as vaginal or anal palpation [29] or using manometry [32] or dynamometry [33].
- Establish any differences between the right and left side during an intra-vaginal digital palpation while the patient contracts and relaxes the PFMs.

**Fig. 3:**
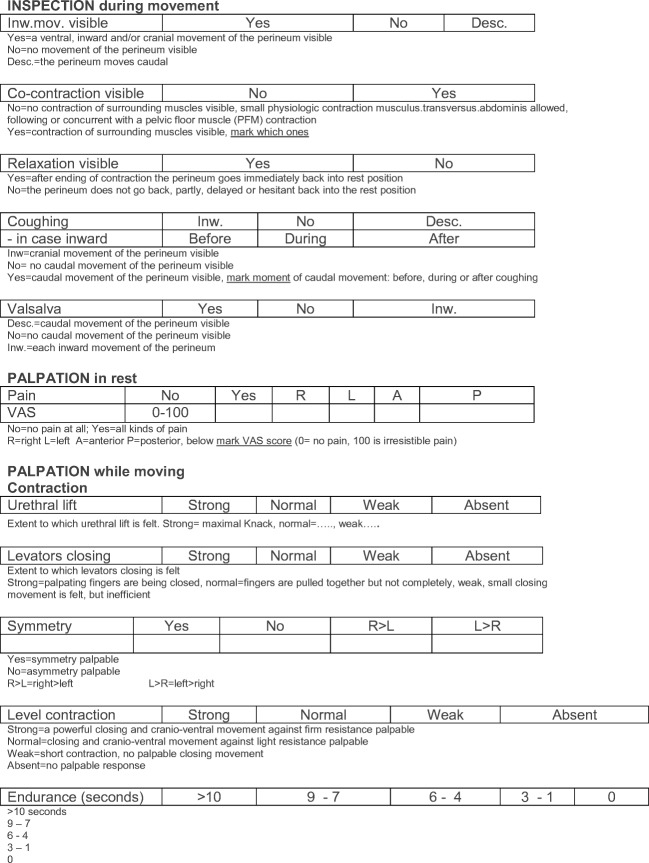

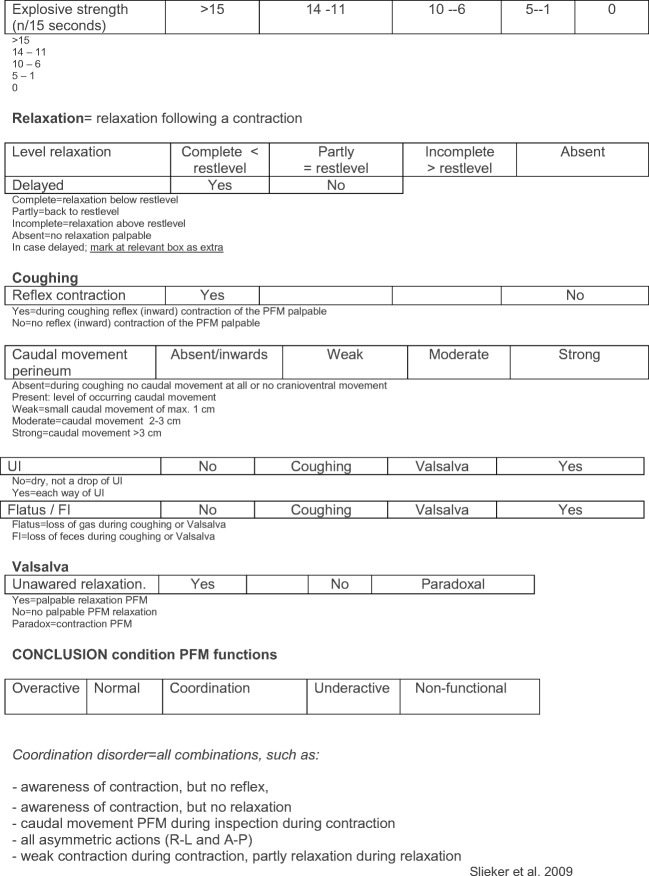
Interpretation scores: pelvic floor muscle functional assessment form. *PFM* pelvic floor muscles, *VAS* visual analog scale, *UI* urinary incontinence, *FI* fecal incontinence

Information about verbal instructions during the pelvic floor muscle functional assessment (PFMFA) and how to interpret scores of the assessment can be found in Fig. 3.

## Interpretation of pelvic floor muscle function

After finishing the PFM functional assessment, the overall interpretation can be made:

- Normal PFMs are indicated by both voluntary and involuntary contraction and relaxation; the voluntary contraction is “normal” or “strong” and the voluntary relaxation is “complete”.
- Overactive PFMs:
  - No or limited relaxation occurs; there is contraction even when relaxation is functionally needed, for instance, during micturition or defecation
  - Symptoms: micturition dysfunction, urine loss, obstructed defecation or dyspareunia
  - Signs: absence of voluntary PFM relaxation, inability to perform a vaginal palpation
- Underactive PFMs:
  - No or insufficient voluntary contraction occurs when it is appropriate
  - Symptoms: UI, anal incontinence
  - Signs: the absence of voluntary and involuntary contractions of the PFMs; possibly pelvic organ prolapse, anal incontinence
- Non-functioning PFMs:
  - PFM action not measurable, neither on instruction to contract (inability) nor as the absence of an automatic response to an increase in intra-abdominal pressure
  - Symptoms: any symptom associated with a non-functioning pelvic floor
  - Signs: any sign of a non-contracting, non-relaxing pelvic floor, possibly avulsion of the levator ani muscle, functional obstructed defecation
- Pain
  - PFM activity may be hindered
  - Symptoms: dyspareunia, vaginism
  - Signs: absence of PFM relaxation, inability to perform a vaginal palpation

## Limitations and potentials of current examination techniques

A limitation of the different measurement methods common to all clinic-based measurements of PFM function is that they are typically performed in the (semi-)supine position. One should keep in mind that this might not reflect functional or usual activity of the pelvic floor during daily life activities as a response or a feed-forward mechanism to increased abdominal pressure [34].

In healthy, continent women, activation of the PFMs before or during physical exertion seems to be an automatic activity; thus, it is, in essence, an unconscious or involuntary contraction [35]. This PFM “reflex” contraction is a result of a fast feed-forward mechanism, resulting in a urethral pressure rise that precedes the bladder pressure rise by 210–270 ms, something that might have been lost in women with UI [35]. A well-timed, fast, and strong PFM contraction might prevent urethral descent during intra-abdominal pressure rise [36, 37]. Therefore, PFM exercises are especially focused on adequate timing, speed of contraction, strength improvement, endurance, adequate relaxation, and coordination of the peri-urethral and PFMs [38]. Appropriate treatment with PFM exercises should always be preceded by an assessment of PFM contraction and relaxation, because the effect of PFM exercises is dependent on whether the contractions and relaxations are performed correctly [36]. The physiotherapy assessment allows the use of individualized exercises, beginning with each patient’s unique capabilities and physical impairments.

As timing of the PFM contraction might be one of the most important elements, this element needs to be included in the assessment.

Instrumental biofeedback with an intra-vaginal or intra-anal probe may serve this objective. For diagnostic use, biofeedback refers to a range of audiovisual techniques whereby information regarding PFM contraction and relaxation is displayed. Electromyography (EMG) is one means by which to do this. Usually, for assessment with EMG biofeedback, the motor unit activity of the PFMs at rest, during a maximal pelvic floor voluntary and involuntary (coughing and Valsalva) contraction, and level of relaxation after maximal contraction are measured. The PFMFA with (preferably wireless) EMG biofeedback incorporates structured assessment in different positions, movements, and activities. Pre-contraction, timing, and coordination of the PFMs are tested.

## Summary of the assessment

The assessment stage involves the explicit decision as to whether “physiotherapy” is the treatment indicated for the patient, based on the findings of the physiotherapy assessment and supplemented by any medical information that accompanied the referral and evaluation. To this end, the physiotherapist has to answer the following questions [2, 30]:

- Does the patient suffer from SUI, UUI, or MUI and UI-related health problems?
- How severe is the UI?
- Are the PFMs dysfunctional? If so, what is the nature of the dysfunction?
- What are the possible causes of this dysfunction?
- Are there currently any local prognostic factors that can adversely affect the recovery and/or adjustment processes, and can these local impeding factors be modified by physical therapy?
- Are there currently any general prognostic factors that can adversely affect the recovery and/or adjustment processes, and can these general impeding factors be modified by physiotherapy?

Once the assessment has been completed and treatment targets have been identified, physiotherapy intervention can begin.

The entire physiotherapy assessment for female UI has been filmed and is available from the first author on request.

## Appendix 1. Example of one of the search strings used

(“Stress incontinence” OR “urgency incontinence” OR (“urge” AND “incontinence”) OR (“mixed incontinence”) OR “urinary incontinence” OR (“urgency” AND “incontinence”)) AND ((“biofeedback” OR (“neuromuscular” AND “conditioning”) OR “conditioning”) OR (“pelvic floor” OR (“pelvic” AND “floor”) OR “pelvic floor”) OR (“electric stimulation” OR (“electric” AND “stimulation”) OR “electrostimulation” OR (“electrical” AND “stimulation”) OR “electrical stimulation”) OR (“kinesiotherapy”/exp. OR exercise/exp. OR physiotherapy/exp. OR “conservative treatment”/de OR ((movement* OR motion OR manual OR phys* OR conservative* OR nonsurg* OR non-operative OR non-surgical OR paramedic* OR para-medical OR exercise) NEAR/5) OR kinesiotherapy* OR gymnastic* OR ((muscle* OR muscul*) NEAR/3 train*))) AND (diagnostic* OR assessment* OR management*) AND ((“random allocation” OR (“random” AND “allocation”) OR “random allocation” OR “randomized”) OR (“guidelines” OR “guideline literature as topic”) OR (“systematic review” OR “review literature as topic”)) AND ((Randomized Controlled Trial[ptyp] OR systematic[sb]) AND full text[sb] AND (“1998/01/01”[PDat]: “2019/11/11”[PDat]) AND Humans[Mesh] AND English[lang] AND Female[MeSH Terms] AND adult[MeSH]) AND ((Randomized Controlled Trial[ptyp] OR systematic[sb]) AND full text[sb] AND (“1998/01/01”[PDat]: “2019/11/11”[PDat]) AND Humans[Mesh] AND English[lang] AND Female[MeSH Terms] AND adult[MeSH]) AND ((Randomized Controlled Trial[ptyp] OR systematic[sb]) AND full text[sb] AND (“1998/01/01”[PDat]: “2019/11/11”[PDat]) AND Humans[Mesh] AND English[lang] AND Female[MeSH Terms] AND adult[MeSH])

## Appendix 2. Search terms

Publications on the nature, seriousness, and importance of stress urinary incontinence (SUI), urgency urinary incontinence (UUI), and mixed urinary incontinence (MUI) as health problems were retrieved using the following search terms: “incidence,” “prevalence,” “aetiology,” “risk factors,” “predictors,” “incontinence,” urinary incontinence,” “detrusor instability,” “detrusor overactivity,” “bladder,” “overactive bladder,” “stress urinary incontinence,” “urge urinary incontinence,” “urgency urinary incontinence “mixed incontinence,” “urgency,” “frequency,” “nocturia,” “involuntary leakage of urine,” “peri- and postpartum dysfunction,” “ pelvic floor dysfunction,” “pelvic floor disorders,” “bladder neck mobility,” “vaginal pressure,” “intra-abdominal pressure,” “previous sexual abuse,” “comorbidity,” “low back pain,” “COPD,” and “depression.”

Publications about the way to diagnose SUI, UUI, MUI, and about the reliability, validity, and feasibility of the diagnostic examinations for physical therapy in routine practice were retrieved by combining the search term “urinary incontinence” with the following key words: “diagnosis,” “assessment,” “guidelines,” “pelvic floor assessment,” “strength,” “endurance,” “pad test,” “diary,” “ultrasound,” “manual muscle testing,” “vaginal squeeze pressure,” “palpation,” “digital assessment,” “manometry,” “pelvic floor function,” “pelvic floor assessment,” “EMG,” “validity,” and “reliability.”

Publications about the types of treatment that are effective and efficient in relation to the nature and severity of SUI, UUI, and MUI as health problems were retrieved by combining “urinary incontinence” with: “physiotherapy,” “physical therapy,” “conservative management,” “conservative therapy,” “conservative treatment,” “life style,” “non-surgical stimulation,” “electrostimulation,” “transcutaneous electrical nerve stimulation,” “neuromuscular stimulation,” “electrical stimulation,” “electrotherapy,” “myofeedback,” “biofeedback,” “pelvic floor,” “pelvic floor muscle training,” “pelvic floor rehabilitation,” “pelvic floor exercises,” “pelvic floor re-education,” “(Kegel) exercises,” “RCTs,” “controlled trials,” “evaluation,” “effectiveness,” “efficacy,” and “outcome.”

## Appendix 3

Guidelines:

1. Nambiar et al. [39]
2. Matsuoka et al. [40]
3. Woodford and George [41]
4. Royal Dutch Society for Physiotherapy (KNGF) [2]
5. Agency for Health Care Policy and Research [42]
6. Fritel et al. [43]
7. Rogers [8]
8. Salmon et al. [44]
9. Tse et al. [5]

Reviews:

1. Mateus-Vasconcelos et al. [45]
2. Lamerton et al. [46]
3. Monteiro S et al. [47]
4. Moser H et al. [38]
5. Deegan et al. [48]
6. Isherwood and Rane [49]
7. Morin et al. [50]

## Appendix 4. Problem categories

1. UI with PFM dysfunction:
  - The patient is unable to identify their PFMs, has no awareness, cannot manage contraction or relaxation; shows no effective involuntary contraction of the PFMs associated with increased abdominal pressure (SUI), or associated with urgency (UUI) or both (MUI) or urgency associated with increased abdominal pressure (stress-induced urgency).
  - The patient is unable to identify their PFMs, has no awareness, cannot manage contraction or relaxation; shows some involuntary contraction of the PFMs associated with increased abdominal pressure (SUI), or associated with urgency (UUI) or both (MUI), or urgency associated with increased abdominal pressure (stress-induced urgency), but the contraction is ineffective.
  - The patient is unable to identify their PFMs, has no awareness, cannot manage contraction or relaxation, but shows effective involuntary contraction of the PFMs associated with increased abdominal pressure (SUI) or associated with urgency (UUI) or both (MUI).
  - The tone of the PFMs is measurably too high, and the patient is unable to reduce this on demand (with or without involuntary tightening and with or without effective involuntary contraction associated with increased abdominal pressure; SUI) or associated with urgency (UUI) or both (MUI).
  - The patient is able to tighten and relax the pelvic floor, but has no effective involuntary control over the pelvic floor associated with increased abdominal pressure (SUI) or associated with urgency (UUI) or both (MUI) or urgency associated with increased abdominal pressure (stress-induced urgency).
  - The patient has both voluntary and involuntary control over their PFMs, but their PFMs are too weak (SUI) or urgency associated with increased abdominal pressure (stress-induced urgency).
  - The functioning of other parts of the musculoskeletal system, for instance, in relation to respiration, voiding posture, and toileting behavior, adversely affects the PFM function (SUI) or urgency associated with increased abdominal pressure (stress-induced urgency).
2. UI without PFM dysfunction, without urgency (SUI), or with urgency (UUI)
3. UI (SUI, UUI, MUI) plus local and/or other (generally) unfavorable prognostic factors that may have adverse local or general effects on recovery and/or adjustment processes, and which may or may not be modifiable by physiotherapy interventions. It is essential to include in the analysis those local and/or other (generally) unfavorable prognostic factors that may have adverse local or general effects on recovery and/or adjustment processes. If such factors cannot be modified, they adversely affect the potential outcome of the physiotherapy intervention. If the factors can be modified by physiotherapy, one of the objectives of the therapy should be to reduce their influence. It would constitute a malpractice to ignore this objective. It is not always possible to decide whether and to what extent physiotherapy is indicated for a particular patient. This is because the diagnostic options available to the physiotherapist may not always provide decisive information about the modifiability of the disorder and/or disease process underlying the SUI. This is particularly true for any local factors that adversely affect the recovery process. The pelvic physical therapist should discuss any doubts about the severity and nature of the disorder and any related health problems with the referring doctor, and/or should refer the patient for further diagnostics and/or review of the management.
